# Testing Whether and When Abstract Symmetric Patterns Produce Affective Responses

**DOI:** 10.1371/journal.pone.0068403

**Published:** 2013-07-01

**Authors:** Marco Bertamini, Alexis Makin, Anna Pecchinenda

**Affiliations:** 1 Department of Psychological Sciences, University of Liverpool, Liverpool, United Kingdom; 2 Department of Psychology, Università di Roma La Sapienza, Rome, Italy; University of Lincoln, United Kingdom

## Abstract

Symmetry has a central role in visual art, it is often linked to beauty, and observers can detect it efficiently in the lab. We studied what kind of fast and automatic responses are generated by visual presentation of symmetrical patterns. Specifically, we tested whether a brief presentation of novel symmetrical patterns engenders positive affect using a priming paradigm. The abstract patterns were used as primes in a pattern-word interference task. To ensure that familiarity was not a factor, no pattern and no word was ever repeated within each experiment. The task was to classify words that were selected to have either positive or negative valence. We tested irregular patterns, patterns containing vertical and horizontal reflectional symmetry, and patterns containing a 90 deg rotation. In a series of 7 experiments we found that the effect of affective congruence was present for both types of regularity but only when observers had to classify the regularity of the pattern after responding to the word. The findings show that processing abstract symmetrical shapes or random pattern can engender positive or negative affect as long as the regularity of the pattern is a feature that observers have to attend to and classify.

## Introduction

Beauty can be described as a social construction, but evolution has also shaped what humans find beautiful. The complex interplay of factors that contribute to subjective beauty makes the empirical study of aesthetics difficult, and some authors are pessimistic. Kubovy, for instance, notes that complex works of art cannot be taken out of a culture, and simple stimuli are too impoverished [1].

Studying novel meaningless stimuli (i.e., shapes seen for the first time) may help to circumvent the problem of familiarity and cultural bias, but it is necessary to find a way to vary the hedonic value of these stimuli in a systematic way. An important question is whether objective dimensions exist that determine positive or negative responses when novel stimuli are presented to naïve observers, and whether these responses are automatic or depend on a process of evaluation.

Symmetry provides a unique opportunity to tackle this problem. There are good reasons to believe that symmetry per se is perceived as pleasant, and is linked to beauty [2]. Symmetry has a central role in visual art as it is present in most cultures and across centuries [3,4]. Symmetry is also present in many animal displays, which makes it unlikely to be exclusively a cultural phenomenon [5–7]. In terms of psychophysics, much is known about the fast and efficient processing of symmetry and in particular of reflectional (or bilateral) symmetry [8–11]. A direct link between symmetry and perceived "goodness" has been shown in the studies by Garner [12] and Palmer [13]. Finally, in 1995, Latto coined the term “aesthetic primitive” to refer to properties that are intrinsically interesting, even in the absence of narrative meaning [14]. Symmetry may be an excellent example of an aesthetic primitive.

Because symmetry can be instantiated in an infinite number of configurations, symmetry offers the opportunity to create and present observers with an infinite number of meaningless novel shapes. If we restrict the discussion to 2D shapes, symmetry depends on the presence of certain rigid transformations, such as translations rotations and reflections. Not all symmetric shapes are the same and their differences can be classified precisely. We will use the term regularity to distinguish these types of shapes because it is more general and because in the literature the term symmetry is often reserved to refer (improperly) to reflectional symmetry.

As first noted by Mach, reflection, especially around a vertical axis, is uniquely salient to human observers when compared to other regularities such as translations and rotations [15]. This has been confirmed by many studies, and much is known about the processing of reflectional symmetry [8–11,16,17]. For instance, Royer compared response time for detection of configurations similar to those we used and found that detection of 90^0^ rotation took almost twice as long as detection of reflection [18]. Rotation is interesting because it is regular in a way that is not easy to detect for human observers, but on terms of information content the same redundancy is present for our Reflection and our Rotation patterns. In other words in both cases one of the four quadrant is sufficient to describe the whole configuration as the other quadrants are the outcome of a rigid transformation.

Novel stimuli characterized by different types of regularities may affect the observer’s hedonic experience because of how they are processed. Many authors have suggested that visual art often finds ways to optimally stimulate the visual system [14,19–22]. More recently, a specific hypothesis has been put forwards about a link between processing fluency and aesthetic preference. Specifically, the fluency hypothesis says that a hedonic response (i.e. a feeling of pleasantness) is directly related to the degree of fluency of processing that information [23,24]. There is some evidence that the hedonic experience engendered by processing fluency emerges in explicit preferences but also in indirect, expressive measures of affect, like smiling [25].

Two types of arguments have been put forward to explain symmetry preference. Firstly, symmetry can serve to evaluate mate or food quality [6,7]. Secondly, if the visual system is tuned to extract symmetry as part of image analysis and object recognition, then symmetry preference may be a by-product of the process by which symmetry is detected [26,27]. For instance the fact that the visual system can extract this property quickly and efficiently may imply an experience of processing fluency. But at a more basic level, it has also been suggested that because the reward network extends to visual areas of the cortex, pleasure may directly originate from visual stimulation [28]. It is therefore important to test how positive affect originates from very brief presentations of visual stimuli, and in situations where no aesthetic evaluation is required.

**Figure 1:**
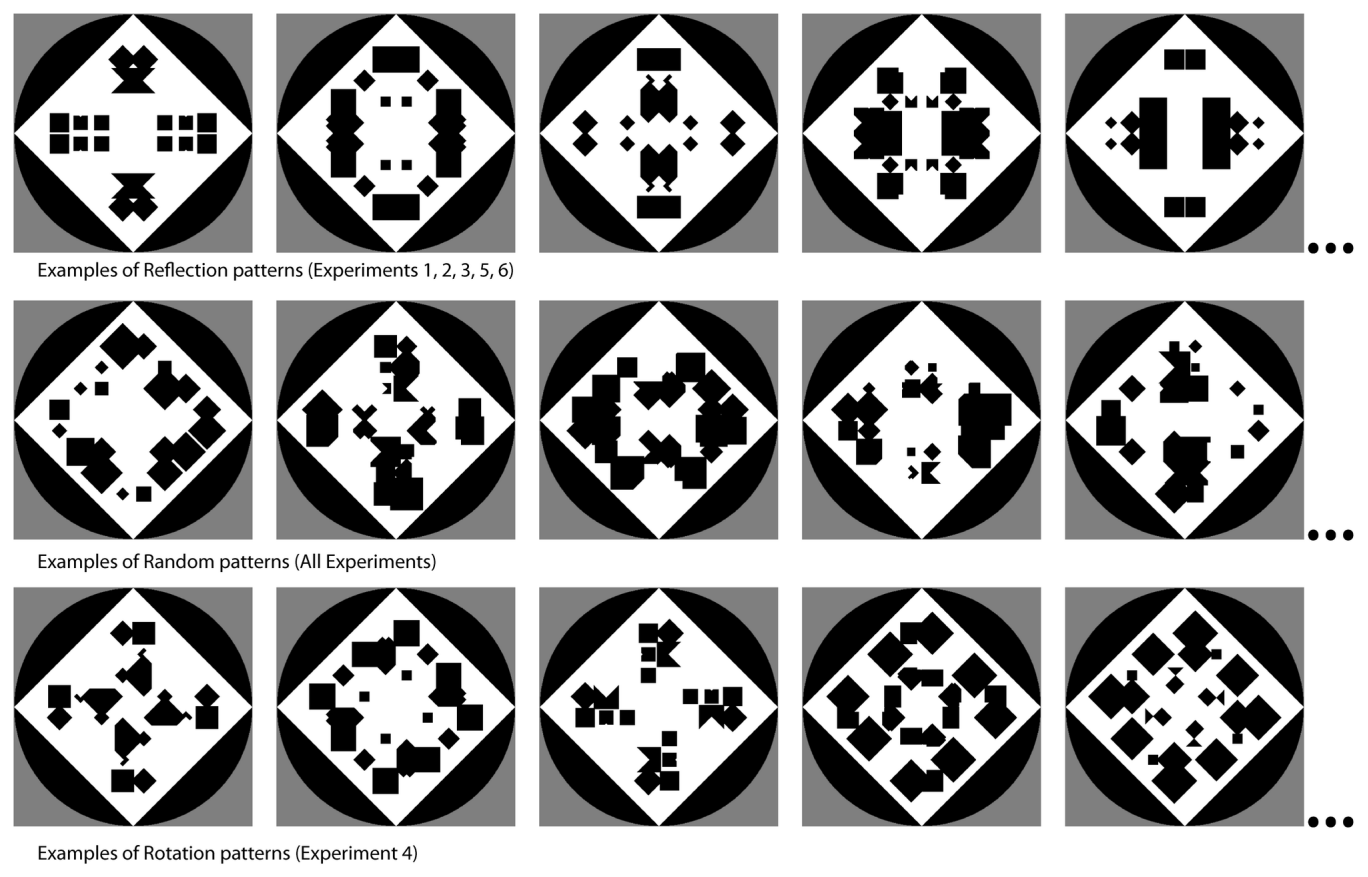
Examples of configurations used as stimuli. Although only five examples are shown here new configurations were created for each trial so that the same pattern was never presented twice.

In our experiments we used configurations of black and white rectangles. They are similar to stimuli used by [29]. Figure 1 shows examples of regular and random patterns. We hypothesized that people would prefer the symmetrical patterns to the random ones. However, participants may find explicit measures of preference unnatural when the stimuli are meaningless. Even the pioneering work by Fechner on the golden ratio has been criticised because in his task observers expressed aesthetic preferences for simple rectangles (for a discussion [30]). Our solution to this problem is to use an implicit measure of affect. To test automatic affective responses to symmetry we used a variant of the object-word interference paradigm [31–34]. In the object-word interference paradigm, two stimuli – a distracter and a target – are presented together or in rapid succession. They can be affectively congruent (both distracter and target denote something good/bad) or affectively incongruent (the distracter denotes something good and the target denotes something bad or vice versa). Typically, response times to targets preceded by congruent stimuli are faster than response times to targets preceded by incongruent stimuli. This finding has been reported with different types of stimuli, including pictures and words.

We hypothesized that people would be quicker to classify word valence on congruent trials (random pattern and negative word, or symmetrical pattern and positive word) than incongruent trials (random pattern and positive word, or symmetrical pattern and negative word). Experiments 1 and 2 test this directly with the only difference that in Experiment 1 observers had to also classify the pattern after responding to the word. We expect this to be an important factor because symmetry may produce positive associations only when it is attended to.

In Experiment 1 after presentation of the pattern (prime) observers had to classify a word as positive or negative. They were asked to respond as quickly and accurately as possible. After responding to the word they had also to report on the presence of symmetry in the prime. This addition to the standard priming paradigm was used to force observers to attend to the pattern, and to force the processing of the symmetry information. To test the importance of this manipulation we removed the second task in Experiment 2. If the priming effect of regularity will be similar in Experiments 1 and 2 this would support the view of an automatic affective response.

## General Methods

### *Participants*

All participants involved in these experiments had normal or corrected-to-normal vision. They were naïve with regards to the specific hypotheses of the study. Each study involved a new sample of participants, but this did not prevent some people from being involved in more than one experiment.

### *Stimuli and Procedure*

Stimuli were generated using psychopy version 1.73 [35] and presented on a CRT monitor with a resolution of 1280 by 1024 pixels at 60 Hz. Each observer sat in front of the monitor in a dimly illuminated and quiet room, at a distance of approximately 57 cm from the screen.

The Random patterns were obtained by placing nine square elements, four of which were black and five were white, in each of four quadrants. The size of the elements varied between 0.25 and 1.0°, and the orientation was either 45 or 90°. The position nearest the central fixation cross was white so the fixation cross was never occluded. The black circle in the background had a diameter of 5.1° at a distance of 57 cm from the screen, similar to the stimuli used by Höfel and Jacobsen [36]. Given this procedure to generate the Random patterns, regularity was introduced by constraining the relationship between quadrants. That is, a rigid transformation (reflection) was used to generate the four quadrants. Therefore, the pattern within each quadrant, taken on its own, was generated by the same procedure (whether the pattern was Random or Reflection) and the regularity was only a property of the relationship between quadrants. This was also true for the Rotation patterns used in Experiment 4. For more details on the stimuli see also 37.

As can be seen in Figure 1, the construction of the patterns based on a fixed procedure implies that there was some variability from trial to trial in terms of black/white ratio and luminance. It is also true that the Random configurations had a degree of regularity due to the split of the pattern in four quadrants. Moreover the regularity of the Random configurations could vary from one example to the next. These are potential problems that were balanced against the advantages of this procedure. Firstly, each quadrant of our stimuli, whether in the random or regular condition, was one instance sampled from the same (vast) population of patterns defined by a specific procedure. Secondly, there was no intervention at any stage by the experimenter in the selection of which patterns to include or exclude.

One hundred and forty-four words were selected from the Affective Norms for English Words (ANEW) database [38], of which 72 were negative (M= 1.90) and 72 were positive (M= 8.17) based on valence ratings (scale 1 to 9). Negative and positive words were matched on arousal (M= 5.95 and M= 6.09 respectively, scale 0 to 9). Although frequency and arousal level were matched (p > 0.26) the two sets were significantly different in terms of valence (p < 0.001). Table S1 shows the complete list of words and their ratings. Each word was only shown once during the experiment.

**Figure 2:**
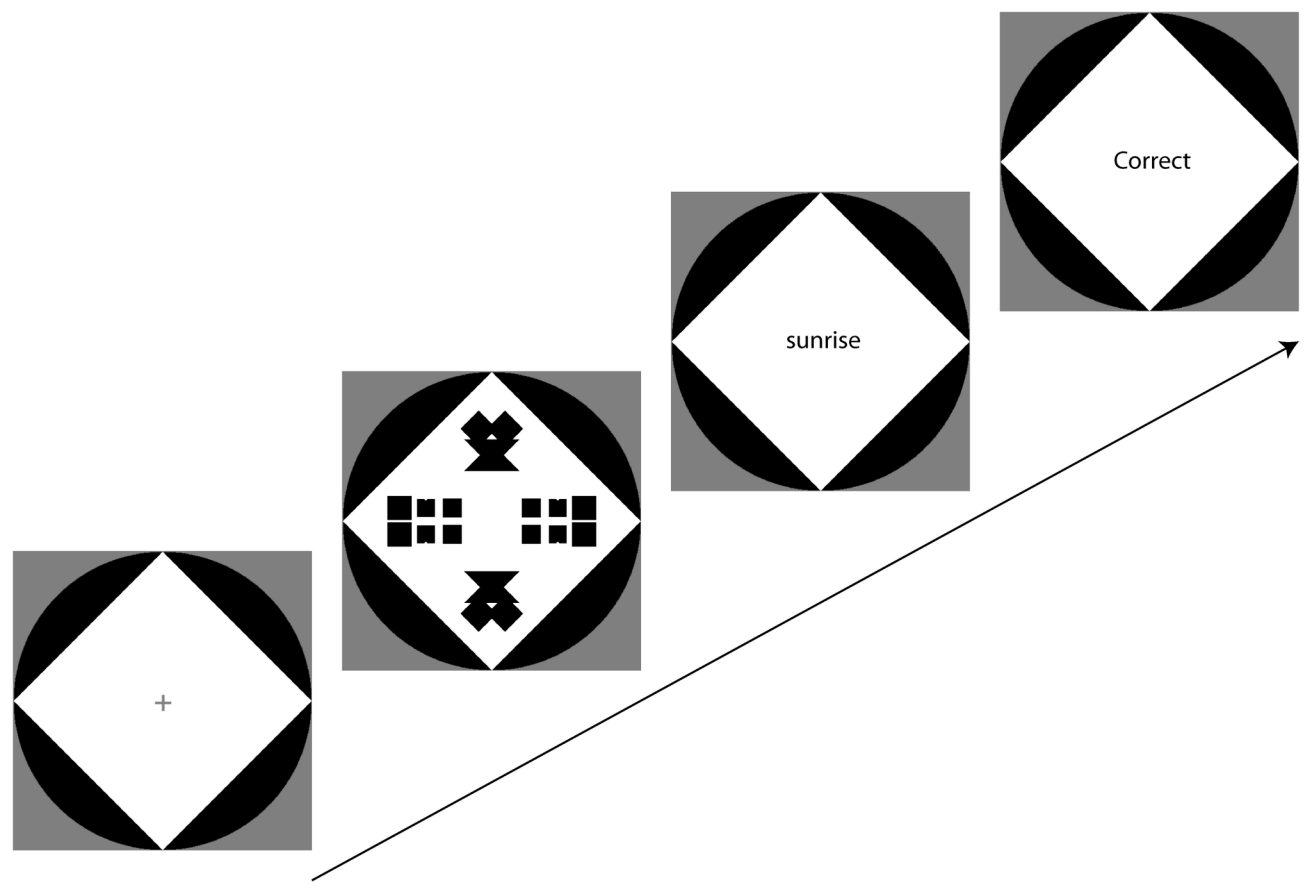
Procedure. A trial started with a fixation mark. A pattern was presented for 250 ms, and after that a word was presented in the centre of the screen. Participants classified the word as quickly as possible as positive or negative by pressing one of two keys. Words are shown larger in this illustration compared to the size used in the experiment.

Figure 2 illustrates the procedure. A trial started with a fixation mark that lasted between 500 or 1150 ms to make the start of a trial less predictable. This was followed by a pattern presented for 250 ms, and immediately afterwards a word was presented in the centre of the screen. Participants had to press the keys ‘k’ or ‘l’ with the right hand to classify the word as either positive or negative. After the word was classified feedback (‘correct’ or ‘incorrect’) was always provided on screen (lasting for 500 ms). Which key was associated with positive valence was counterbalanced for different participants. Participants did not take part in more than one experiment so as to remain naïve.

In each experiment observers first completed a block of 20 practice trials, followed by an experimental block with a total of 144 trials in random order, with a rest break in the middle. The patterns and the target-words used in the practice block were not included in the experimental block.

### *Ethics Statement*

All experimental procedures were approved by the Ethics Committee of the University of Liverpool prior to the commencement of the study. Informed written consent was obtained from all participants.

### *Analysis*

For each experiment we analysed the response time in the word classification task and, separately, error rates for the speeded classification task. The factors included in the repeated-measures ANOVA were valence (positive or negative) and regularity (symmetrical pattern or random pattern). Errors were excluded in the ANOVAs for response time. We will only report analyses based on means, but we also carried out the analysis on the medians and the same pattern is present for both. When comparing experiments equal variances were not assumed and degrees of freedom adjusted. For ANOVA we report partial η^2^, which gives the proportion of variance explained by the factor (after the variance due to the other factors and interactions has been partialed out). As a simple rule of thumb the criterion introduced by Cohen for small (0.01) medium (0.06) and large (0.14) effect sizes can be used.

## Experiment 1: Reflectional Symmetry

In this experiments observers were presented with novel visual pattern for 250 ms and immediately afterwards they had to classify a word as either positive or negative. After the response to the valence of the word, observers had to remember and classify the pattern as either random or symmetrical.

**Method**

*Participants.* Participants were 24 undergraduate students at the University of Liverpool (age 18-31; 4 males).

*Stimuli and procedure.* Stimuli and procedure are described in the General Methods section. After the response to the valence of the word, the text “Was it symmetrical?” appeared and observers had to remember and classify the pattern using the keys ‘a’ and’s’ with their left hand.

**Results**

**Figure 3:**
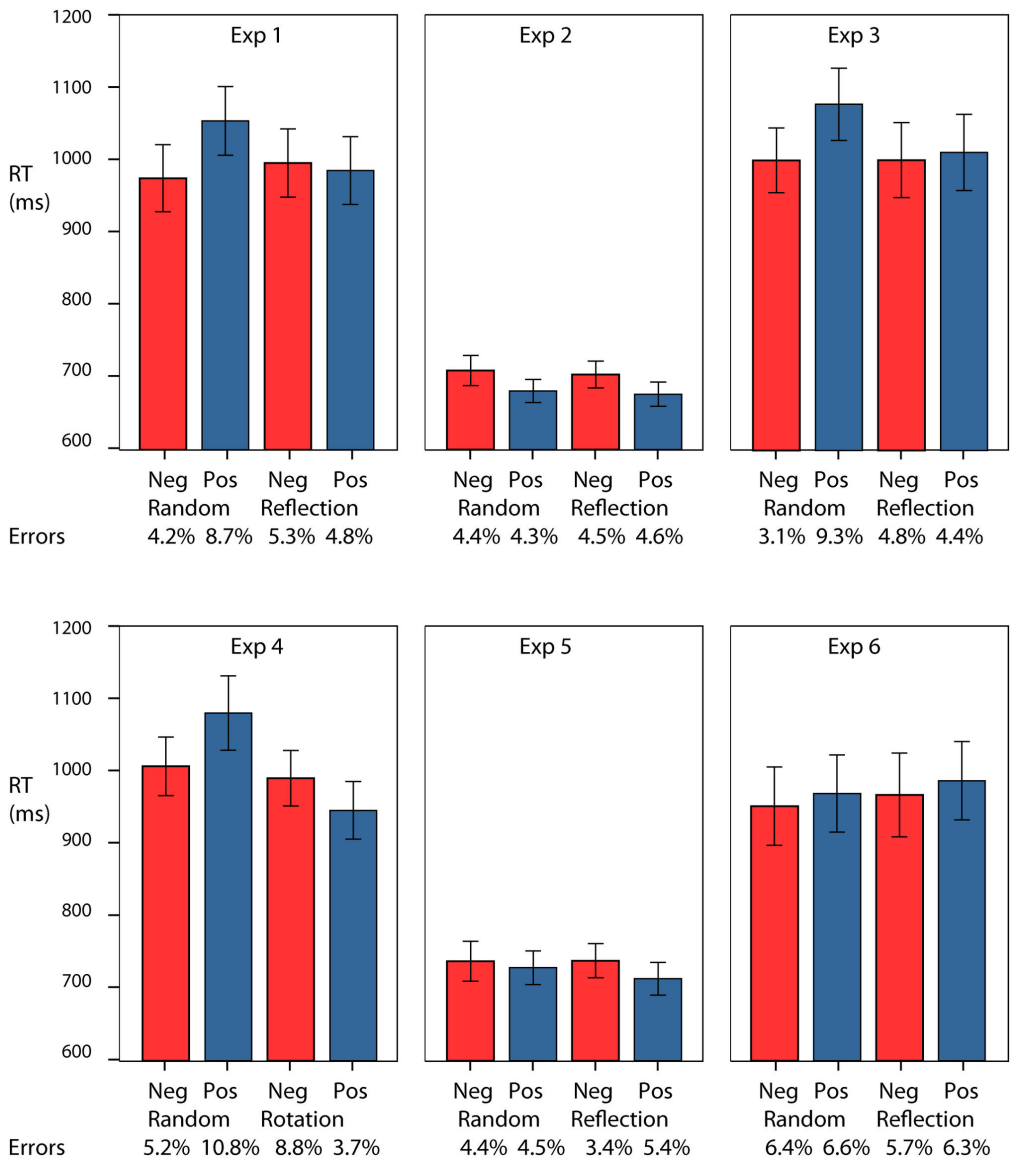
Mean RT and error rates for Experiments 1-6. The error bars are ±1SEM.

Mean RT are shown in Figure 3. Trials were excluded if there was an error on either the word classification or the reporting of the regularity (18%). There was a main effect of valence (F(1,23)=5.46, p<0.05; partial η^2^=0.19): responses were faster for negative words, and an interaction between regularity and valence (F(1,23)=10.52, p<0.01; partial η^2^=0.31): responses were faster for negative words after a Random pattern and faster for positive words after a Reflection pattern. The interaction appears driven by a significant difference in speed of responding to the positive or negative words after presentation of the random pattern (t(23)=-3.66, p<0.01) rather than the regular pattern (t(23)=0.56, n.s.). However, such differences need to be interpreted with caution without a baseline. We will address this issue with the last experiment.

The analysis of error rates found no effect of valence or regularity, but there was an interaction between regularity and valence (F(1,23)=4.87, p<0.05; partial η^2^=0.17). Most importantly the pattern was consistent with the response time data and, therefore, there was no evidence of any speed accuracy trade-off. Mean error rates for each condition are shown below the graphs in Figure 3.

## Experiment 2: Reflectional Symmetry without Pattern Classification

Experiment 2 was identical to Experiment 1 except that observers did not have to report any information about the patterns. Experiment 2 is closer to the pure affective priming procedures [39].

**Method**

*Participants.* Participants were 24 undergraduate students at the University of Liverpool (age 18-24; 4 males).

*Stimuli and procedure.* The stimuli were the same as those used in Experiment 1, however, after the feedback screen observers were not asked a question about the symmetry of the pattern.

**Results**

Mean RT are shown in Figure 3. There was a main effect of valence (F(1,23)=5.60, p<0.05; partial η^2^=0.19): responses were faster for positive words, but there was no interaction between regularity and valence (F(1,23)=0.01, n.s).

The same analysis on error rates (5% overall) found no significant effect of valence or regularity, and no interaction between regularity and valence. There was no evidence of a speed accuracy trade-off. Mean error rates for each condition are shown below the graphs in Figure 3.

To summarise the congruency effect we computed the difference between congruent and incongruent trials. By design what we call congruent trials are trials in which positive words are preceded by symmetry or alternatively negative words are preceded by random patterns. This allowed us to test the difference between the strength of the congruency effects in Experiments 1 and 2 using a between-subjects t test. This difference was significant, because the affective congruency effect was stronger in Experiment 1 (t(34.33)=2.729, p <0.05).

## Experiment 3: Reflectional Symmetry with a Different Classification Question

It is possible that positive affect is linked with target presence and therefore with a yes response. In a recent study such an effect was found for responses to symmetry measured using electromyography (EMG) [37]. To test the importance of a yes response to the pattern we changed Experiment 1 so that participants were asked whether the patter was Random. A symmetric pattern now requires a no response.

**Method**

*Participants.* Participants were 24 undergraduate students at the University of Liverpool (age 18-28; 3 males).

*Procedure.* The stimuli were the same as those used in Experiment 1, however, observers were asked a different question. The text “Was it random?” appeared and they had to classify the pattern using the keys ‘a’ and’s’ with their left hand. Therefore, Random trials may now be conceptualised as target present trials.

**Results**

Mean RT are shown in Figure 3. Error trials were excluded (19%). There was a marginal effect of regularity (F(1,23)=4.84, p<0.05; partial η^2^=0.17): responses to words were faster after a reflection pattern. There was a main effect of valence (F(1,23)=7.65, p<0.05; partial η^2^=0.25): responses were faster for negative words. There was an interaction between regularity and valence (F(1,23)=8.01, p<0.01; partial η^2^=0.26). Based on a pair of t tests the interaction appears driven by a significant difference in speed of responding to the positive or negative words after presentation of the random pattern (t(23)=-3.61, p<0.01) rather than the regular pattern (t(23)=-0.58, n.s.).

This interaction was therefore consistent with that of Experiment 1 and different from the result of Experiment 2. By comparing Experiments 1 and 3 it emerges that which category served as the target for the second task (symmetry or random) did not produce a substantial change in the results. That is, the congruency effect was of similar magnitudes in Experiment 1 with where reflection was the reported with a yes key, and in Experiment 3 with random was reported with a yes key (t(46) = 0.6, n.s).

The analysis of error rates found marginal effects of valence (F(1,23)=6.27, p<0.05; partial η^2^=0.21) and regularity (F(1,23)=5.25, p<0.05; partial η^2^=0.18), and an interaction between regularity and valence (F(1,23)=17.64, p<0.01; partial η^2^=0.43). The pattern was consistent with the response time data and, therefore, there was no evidence of speed accuracy trade-off. Mean error rates for each condition are shown below the graph in Figure 3.

## Experiment 4: Rotational Symmetry

Experiment 4 was based on Experiment 1 but instead of Reflection we tested a 90^°^ Rotation (see Figure 2). A rotation produces a pattern that is regular and the degree of redundancy is the same as that in the patterns of Experiment 1 in that all four quadrats were related to each other. However, this type of symmetry tends to be less salient for human observers [18].

**Method**

*Participants.* Participants were 24 undergraduate students at the University of Liverpool (age 18-44; 5 males).

*Procedure.* Everything was the same as in Experiment 1, except for the stimuli. The text “Was it symmetrical?” was used as in Experiment 1 and it was explained to the participants that the symmetry in the stimuli was a rotational symmetry.

**Results**

Mean RT are shown in Figure 3. Error trials were excluded (26%). There was an effect of regularity (F(1,23)=13.74, p<0.01; partial η^2^=0.37): responses to words were faster after a rotation pattern, but no main effect of valence (F(1,23)=0.652, n.s.). There was an interaction between regularity and valence (F(1,23)=13.75, p<0.01; partial η^2^=0.37). This interaction was consistent with that of Experiment 1. Based on a pair of t tests the interaction appears driven by a significant difference in speed of responding to the positive or negative words after presentation of the random pattern (t(23)=-3.07, p<0.01) rather than the regular pattern (t(23)=1.83, n.s.).

As with Experiments 2 and 3, we compared the strength of the congruency effect with that of Experiment 1. This difference was not significant (t(46)=0.43, n.s.).

The analysis of error rates found a marginal effect of regularity (F(1,23)=4.98, p<0.05; partial η^2^=0.17) and an interaction between valence and regularity (F(1,23)=18.83, p<0.01; partial η^2^=0.45). The pattern was consistent with the response time data and, therefore, there was no speed accuracy trade-off. Mean error rates for each condition are shown below the graph in Figure 3.

## Experiment 5: Memory

Experiment 5 was the same as Experiment 1, but observers were asked to memorise the patterns for a later memory test. Therefore, as in Experiment 1, observers had to attend to the patterns but there was no immediate question. In Experiment 5 we test whether it is possible to direct attention to the patterns without directing attention necessarily to the symmetry per se, and whether this increased attention can drive the congruency effect.

**Method**

*Participants.* Participants were 24 undergraduate students at the University of Liverpool (age 18-47; 5 males).

*Procedure.* Everything was the same as in Experiment 2, except that it was explained to the participants that after the end of the experiment they had to also perform a memory test for the patterns that they had seen.

**Results**

Mean RT are shown in Figure 3. The overall level of performance was more similar to that of Experiment 2 than that of Experiment 1. Error trials were excluded (5%). There was no effect of regularity (F(1,23)=1.04, n.s.) or valence (F(1,23)=2.88, n.s.), and there was no interaction between regularity and valence (F(1,23)=0.99, n.s.). The lack of interaction contrasts with the finding of Experiment 1. The congruency effect was weaker than in Experiment 1 (t(46) = 2.11, p< 0.05). The analysis of error rates found no significant effects or interactions. Mean error rates are shown below the graph in Figure 3.

Participants found the memory task hard, performance on the post-experiment test was close to chance: 51% correct. Remembering abstract configuration is a difficult task, so perhaps this manipulation did not really encourage people to attend to the patterns. In Experiment 6 we introduced a simple judgment of the pattern that can be performed accurately.

## Experiment 6: Introducing a New Pattern Property

**Figure 4:**
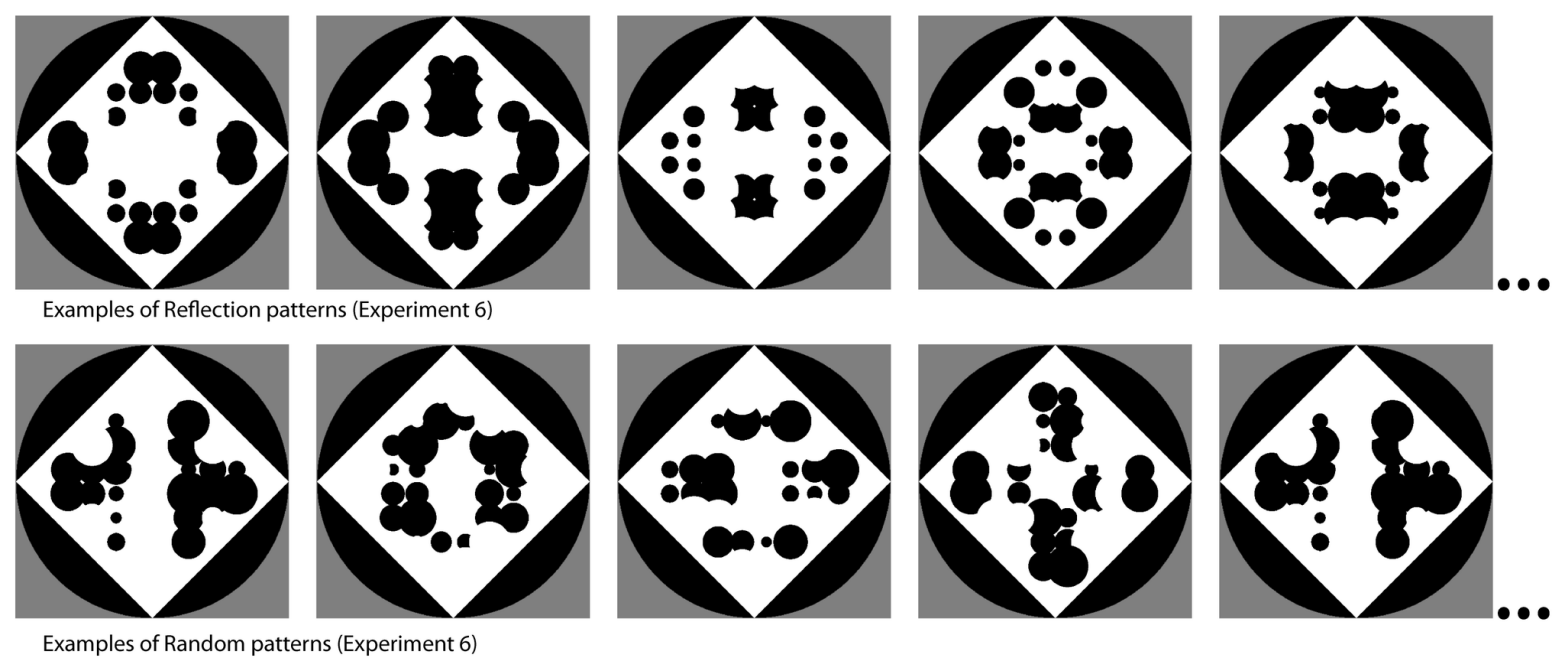
Examples of configurations used as stimuli in Experiment 6. These new configurations created using circular black and whire regions were interleaved with the configurations created using squares which are illustrated in Figure 1.

In this study the patterns were modified and observers had to report a new property of the patterns: whether they were composed of square or circular regions. The former were identical to the stimuli used in Experiment 1, the latter were new. The new set of stimuli was created by replacing the square regions with circles of varying diameter, as illustrated in Figure 4. This new dimension was orthogonal to the presence of regularity, in that for both sets there were symmetrical and random versions. Therefore, as in Experiment 1, observers had to attend and report a property of the patterns after responding to the word. However, unlike Experiment 1, this property was unrelated to the regularity of the pattern. If the results are similar to those of Experiment 1 we can conclude that attention is a key factor and that attention can be directed to the patterns in many ways. If the results are different then categorization rather than attention is the critical factor. Experiment 6 tested a condition in which a property of the patterns is accurately reported.

**Method**

*Participants.* Participants were 24 undergraduate students at the University of Liverpool (age 18-44; 4 males).

*Procedure.* Everything was the same as in Experiment 1, except for the stimuli and the second question. The text “Was it squares?” was used and it was explained to the participants that they had to report which of the two types of stimuli that had seen.

**Results**

Mean RT are shown in Figure 3. The overall level of performance was more similar to that of Experiment 1 than that of Experiment 2. Error trials were excluded (17%). There was no effect of regularity (F(1,23)=3.29, n.s.) or valence (F(1,23)=1.66, n.s.), and there was no interaction between regularity and valence (F(1,23)=0.01, n.s.). The lack of interaction contrasts with the finding of Experiment 1, and accordingly the congruency effect was significantly attenuated or absent (t(46) =2.54, p<0.05)

The analysis of error rates (17% overall) found no significant effects or interactions. Mean error rates for each condition are shown below the graph in Figure 3.

## Experiment 7: No Prime Baseline

The interaction between valence of the word and regularity suggests that, in relative terms, regular and irregular patterns share their valence more with positive and negative words respectively. However, the interaction could be due to just one of the two associations. For instance it is possible that irregular patterns have negative valence but regular patterns are neutral. To separate the two effects we repeated Experiment 1 but included a novel condition, one in which no pattern was shown before the word. Instead the screen remained blank for the interval equivalent to the pattern presentation.

**Method**

*Participants.* Participants were 24 undergraduate students at the University of Liverpool (age 18-34; 8 males).

*Procedure.* Everything was similar to Experiment 1, except that the patterns were divided into three instead of two categories. The new category was one in which there was a blank screen instead of a pattern. In terms of the classification of the pattern, in addition to the two keys that were used in Experiment 1, observers were asked to press the space bar if no pattern had appeared before the word.

**Results**

**Figure 5:**
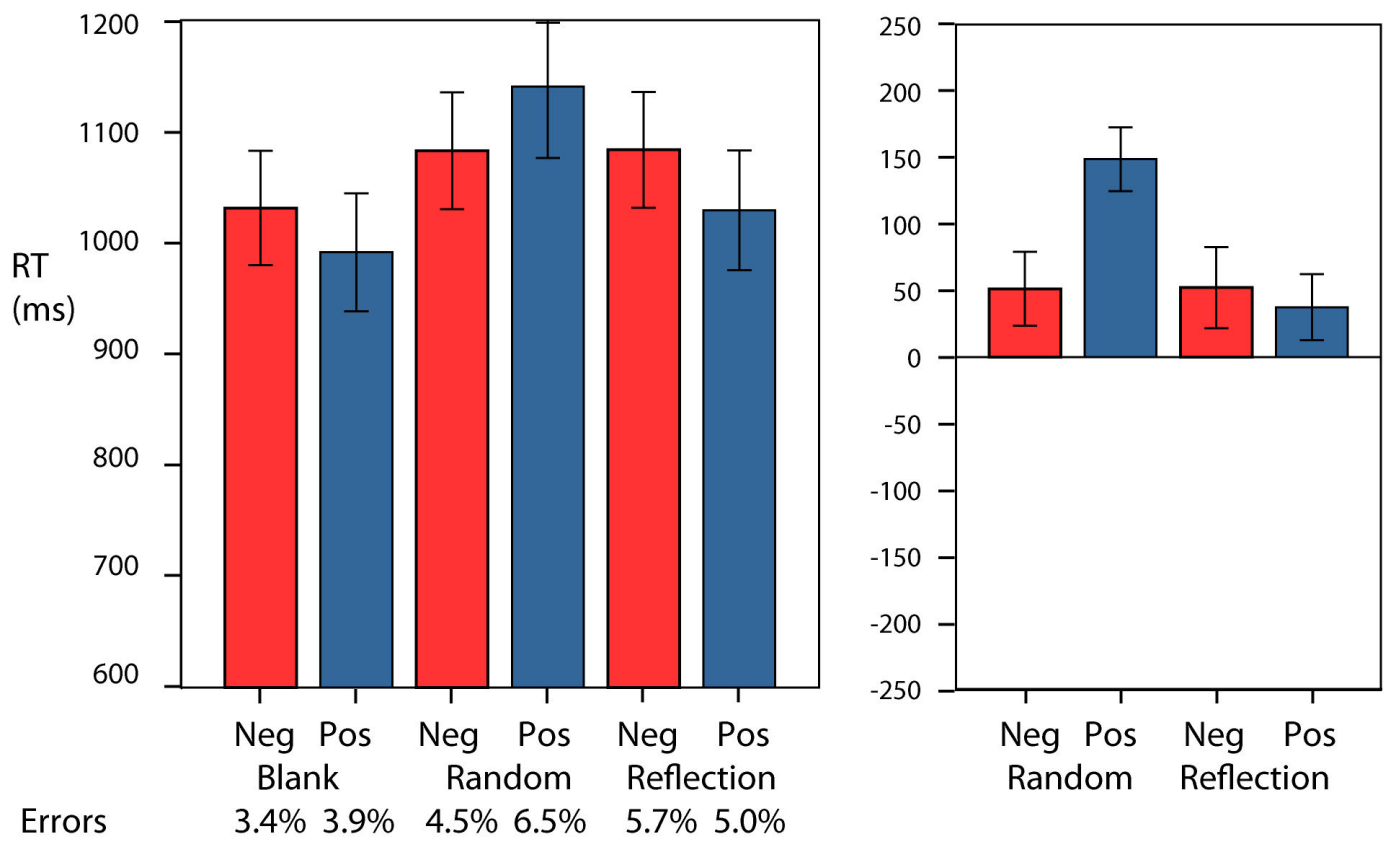
Mean RT and error rates for Experiment 7. On the right the results for Random and Reflection are replotted after subtracting the baseline (Blank) for negative and positive words. Values above zero show the degree to which the responses are slowed in each condition. The error bars are ±1SEM.

Mean RT are shown in Figure 5. The factor regularity had three levels (blank, random, and reflection). Error trials were excluded (12%) whether the error was on the word classification or on the pattern classification. There was an effect of regularity (F(2,46)=13.59, p<0.01; partial η^2^=0.37), no effect of valence (F(1,23)=0.35, n.s.), and there was an interaction between regularity and valence (F(2,46)=5.42, p<0.01; partial η^2^=0.19).

It appears that in the Blank condition responses were faster to positive words. This is consistent with the literature [40] but in itself it is not of interest to the present study. The purpose of the experiment was to compare the effects of Random and Reflection against a baseline provided by the Blank condition. Therefore we computed difference scores using the two means from the Blank condition (for negative and positive words). These are shown in Figure 5. Two additional t tests were used to establish whether there was a significant difference between negative and positive conditions after this adjustment. For Reflection there was no difference (t(23)=0.36, n.s.) but for Random there was a significant difference (t(23)=-3.20, p<0.01).

The analysis of error rates found no significant effects or interactions. Mean error rates are shown below the graph in Figure 3.

## Discussion

The present study investigated to what extent the processing of novel shapes can engender positive affect. In a series of experiments we interleaved irregular black and white patterns with patterns containing reflectional symmetry (Experiments 1-3 and 5-7) or rotational symmetry (Experiment 4). We recorded the latency to classify words with positive or negative affective valence presented after the regular or irregular patterns. To ensure that familiarity did not play a role in our results no pattern and no word was ever repeated during an experiment.

The outcome was clear: when participants had to report the regularity present in the stimulus there was a congruency effect. At least in relative terms, regular patterns were associated with faster responses to positive words and irregular patterns were associated with faster responses to negative words. The results of Experiment 4 extended the study to rotational symmetry, which is less salient than reflectional symmetry [18]. We found that rotational symmetry generated the same effect as reflectional symmetry.

These conclusions have to be qualified by the results of Experiments 2, 5, and 6. When observers are not asked to report the type of pattern as regular or irregular then there is no effect on the speed of the responses to the words. In Experiment 2 one may suggest that this is a ceiling effect as the task was now carried out more quickly in general, but in Experiment 6 the overall speed was similar to that of Experiment 1 and yet there was no modulation based on the pattern type. Experiment 6 differed from Experiment 1 in that observers had to report a characteristic of the pattern, but this was orthogonal to the regularity: Half of the patterns were made with squares and the other half with circles. Observers reported this difference and did not have to report the fact that within each type half were regular and half irregular.

The importance of drawing attention to the regularity in the pattern is in line with some perception studies that have found that focused attention is critical for detection of symmetry. For example, Olivers and van der Helm [41] found that regular patterns do not pop out in visual search tasks. In other work, Jacobsen and Höfel [29] found that Event Related Potential (ERP) responses to symmetry are present in the absence of any explicit symmetry detection task, but that amplitude was reduced in these conditions. Meanwhile, Sasaki et al. [42] report reduced BOLD responses to reflectional symmetry when participants attend to a non-symmetrical aspect of the patterns (as in our Experiment 6). It seems to be the case that visual processing of symmetry is enhanced when attention is focused on symmetry detection, and that affective responses to symmetry emerge only in these attention-focused conditions.

In a recent paper Makin et al. [43] have used the implicit association test (IAT) to measure the emotional response to visual regularities and matched random patterns. This procedure was comparable, but not identical, to the affective congruence task of Experiment 1. Participants saw images and words interleaved trials. On some trials they had to categorize images as reflection or random. On others, they categorized words as positive or negative. When the same button was used to report reflection and positive words, responses were faster than when the same button was used to report reflection and negative words. This classic IAT effect was taken as an implicit measure of preference for reflection over random patterns, in agreement with the results of Experiments 1 and 3 of the current work. Makin et al. also reported the null results of an Extrinsic Affective Simon Task (EAST), in which participants saw similar stimuli to the IAT, but classified the patterns according to colour rather than regularity [43]. There was no evidence for positive responses to regularity or negative responses to random in the EAST, in agreement with the current Experiment 6. In summary, converging evidence suggests that attention to the regular/irregular dimension is a prerequisite for emotional responses to abstract patterns.

This differs subtly but crucially from the conclusions of Höfel and Jacobsen who argue that affective responses to symmetry only occur when people are evaluating the patterns aesthetically – but not when categorizing them in terms of their objective regularity [44,45]. This series of experiments suggests that attention to regularity is sufficient to engender affective responses, although this response is not automatic.

How can we be sure that the affective congruency has anything to do with the visual stimuli, rather than the conceptual categories reflection and random? Put another way, one could ask: Did the image produce an emotional response, which subsequently biased word valence discrimination, or did the conceptual representations and associations produce an emotional response, which subsequently biased word valence discrimination? For instance, Dittrich and Klauer have recently suggested that the devaluation of a distractor stimulus is mediated by the affective connotation implied by the response label (what is labeled as the target) and by the instructions [46].

The current data does not provide an easy answer. However, this kind of question presupposes a modular model of neuro-cognitive systems. Instead, the conceptual categories of symmetry and random could be represented, in part, by visual nodes responsible for visual symmetry detection. Conversely, any activation of the conceptual categories symmetry and random (e.g. by hearing a word or seeing it written on a screen), could enhances visual processing of the regularity of the stimulus [47,48]. Seen this way, our results support the possibility that when the activation of the distributed, multimodal network that represents the regularity of a stimulus is enhanced, this enhancement biases word valence discrimination.

Another important aspect of the results emerged in Experiment 7. This experiment was similar to Experiment 1 but we included, in addition to regular and irregular patterns, a condition that had no pattern. The results suggest that the blank screen produced responses similar to the reflection patterns. The condition in which responses to the words were changed was that with random patterns. This suggests that Random is associated with negative valance and maybe that reflection is not necessarily associated with positive valence. One has to be careful, however, in considering the properties of a blank screen, as a uniform surface can be classed as highly regular. This may have been particularly true in our experiment given that a circular background was always present on the screen (see Figure 1).

Our study bridges two different areas: research showing that some types of symmetry are salient for human observers, and the central role of symmetry in visual arts and aesthetics. The results can also be taken as supporting the fluency hypothesis, which states that positive affect stems from the ease of processing a given stimulus [23]. If symmetric configurations are processed more fluently compared to random configurations, they should engender a positive affect resulting in a greater advantage when reading positive words compared to negative words. This is what we found. Nevertheless, the fluency account would predict reduced affective priming for rotational symmetry, which is less salient for humans [18], which we did not find (Experiment 4). It might be that our procedure was not sensitive enough to pick up on the difference between reflection and rotation, but can tap the larger reflection/random fluency differences. Moreover, Makin et al. [43] compared reflection and rotation directly, and they did find an implicit preference for reflection, in line with the fluency account.

Other work on the fluency hypothesis has focused on the effects of previous stimulus exposure [49,50] or of stimulus visibility [51] on participants’ preferences. In our experiments the visual patterns were meaningless configurations that had never been seen before. We also designed our experiments so that neither a pattern nor a word was ever seen twice by an observer. This means that familiarity cannot explain the findings.

In conclusion, this work clarifies some important issues about aesthetic processing. First, it shows that abstract patterns with no semantic content can produce affective responses, and they are not too impoverished. Of course, human aesthetic creations are far richer than the kind of laboratory-controlled stimuli used in our work [1]. Nevertheless, symmetry has a role as an aesthetic primitive [14] that artists and designers can, and have, exploited for centuries [4,52]. Second, the work shows that affective responses can be elicited automatically, that is, in the absence of any overt instruction to categorize the patterns as beautiful or ugly. Third, and importantly, it shows that this affective processing occurs only when the relevant dimension (in this case regularity) is actively attended.

## Supporting Information

Table S1 The two sets of words used in the experiment, taken from the ANEW database (Bradley & Lang, 1999).
(DOCX)
