# Supplementary material for: Testing Whether and When Abstract Symmetric Patterns Produce Affective Responses
Source: PLoS One. 2013 Jul 1;8(7):e68403. doi: 10.1371/journal.pone.0068403 (PMC3698216; doi:10.1371/journal.pone.0068403)
Supplement: Table S1 — The two sets of words used in the experiment, taken from the ANEW database (Bradley & Lang, 1999). (DOCX) [file pone.0068403.s001.docx]

**Positive Valence Arousal Freq. Negative Valence Arousal Freq.**

win 8.38 7.72 55 accident 2.05 6.26 33

ecstasy 7.98 7.38 6 maggot 2.06 5.28 2

loved 8.64 6.38 56 torture 1.56 6.1 3

millionaire 8.03 6.14 2 vomit 2.06 5.75 3

acceptance 7.98 5.4 49 drown 1.92 6.57 3

liberty 7.98 5.6 46 murderer 1.53 7.47 19

kiss 8.26 7.32 17 disaster 1.73 6.33 26

rainbow 8.14 4.64 4 hurt 1.9 5.85 37

proud 8.03 5.56 50 toxic 2.1 6.4 3

sweetheart 8.42 5.5 9 hate 2.12 6.95 42

delight 8.26 5.44 29 sad 1.61 4.13 35

graduate 8.19 7.25 30 pain 2.13 6.5 88

miracle 8.6 7.65 16 bomb 2.1 7.15 36

wedding 7.82 5.97 32 depression 1.85 4.54 24

victory 8.32 6.63 61 despise 2.03 6.28 7

romantic 8.32 7.59 32 death 1.61 4.59 277

sex 8.05 7.36 84 mutilate 1.82 6.41 3

happy 8.21 6.49 98 war 2.08 7.49 464

sunrise 7.86 5.06 10 headache 2.02 5.07 5

party 7.86 6.69 216 demon 2.11 6.76 9

vacation 8.16 5.64 47 terrible 1.93 6.27 45

comedy 8.37 5.85 39 funeral 1.39 4.94 33

love 8.72 6.44 232 useless 2.13 4.87 17

home 7.91 4.21 547 lost 2.82 5.82 173

baby 8.22 5.53 62 dead 1.94 5.73 174

music 8.13 5.32 216 bankrupt 2 6.21 5

caress 7.84 5.14 1 poison 1.98 6.05 10

beach 8.03 5.53 61 abuse 1.8 6.83 18

handsome 7.93 5.95 40 misery 1.93 5.17 15

fun 8.37 7.22 44 infection 1.66 5.03 8

sexy 8.02 7.36 2 hatred 1.98 6.66 20

terrific 8.16 6.23 5 rape 1.25 6.81 5

satisfied 7.94 4.94 36 terrified 1.72 7.86 7

free 8.26 5.15 260 cancer 1.5 6.42 25

engaged 8 6.77 47 rejected 1.5 6.37 33

pleasure 8.28 5.74 62 burial 2.05 5.08 11

success 8.29 6.11 93 jail 1.95 5.49 21

orgasm 8.32 8.1 7 rabies 1.77 6.1 1

valentine 8.11 6.06 2 stress 2.09 7.45 107

waterfall 7.88 5.37 2 suicide 1.25 5.73 17

confident 7.98 6.22 16 depressed 1.83 4.72 11

joy 8.6 7.22 40 ulcer 1.78 6.12 5

fame 7.93 6.55 18 upset 2 5.86 14

mother 8.39 6.13 216 assault 2.03 7.51 15

lucky 8.17 6.53 21 poverty 1.67 4.87 20

triumphant 8.82 6.78 5 hell 2.24 5.38 95

luxury 7.88 4.75 21 slave 1.84 6.21 30

joke 8.1 6.74 22 morgue 1.92 4.84 1

merry 7.9 5.9 8 paralysis 1.98 4.73 6

paradise 8.72 5.12 12 afraid 2 6.67 57

birthday 7.84 6.68 18 failure 1.7 4.95 89

cash 8.37 7.37 36 distressed 1.94 6.4 4

affection 8.39 6.21 18 disloyal 1.93 6.56 2

champion 8.44 5.85 23 slaughter 1.64 6.77 10

achievement 7.89 5.53 65 suffocate 1.56 6.03 1

thrill 8.05 8.02 5 gloom 1.88 3.83 14

kindness 7.82 4.3 5 anguished 2.12 5.33 2

excellence 8.38 5.54 15 tragedy 1.78 6.24 49

cheer 8.1 6.12 8 nightmare 1.91 7.59 9

humor 8.56 5.5 47 betray 1.68 7.24 4

aroused 7.97 6.63 20 fear 2.76 6.96 127

hug 8 5.35 3 grief 1.69 4.78 10

snuggle 7.92 4.16 4 killer 1.89 7.86 21

diamond 7.92 5.53 8 cruel 1.97 5.68 15

laughter 8.45 6.75 22 alone 2.41 4.83 195

joyful 8.22 5.98 1 trauma 2.1 6.33 1

promotion 8.2 6.44 26 sick 1.9 4.29 51

pillow 7.92 2.97 8 unhappy 1.57 4.18 26

friendly 8.43 5.11 61 loneliness 1.61 4.56 9

treasure 8.27 6.75 4 victim 2.18 6.06 27

passion 8.03 7.26 28 prison 2.05 5.7 42

god 8.15 5.95 318 divorce 2.22 6.33 29

Mean 8.17 6.09 53.18 Mean 1.90 5.95 39.65

Appendix 1. The two sets of words used in the experiment, taken from the ANEW database (Bradley & Lang, 1999). Positive words are on the left and negative words on the right.
